# Remediation Strategies for Performance Anxiety across Sex, Sport and Stage: Identifying Common Approaches and a Unified Cognitive Model

**DOI:** 10.3390/ijerph181910160

**Published:** 2021-09-27

**Authors:** David L. Rowland, Gene Moyle, Stewart E. Cooper

**Affiliations:** 1 Department of Psychology, Valparaiso University, Valparaiso, IN 46383, USA; 2 Faculty of Creative Industries, Education, and Social Justice, Queensland University of Technology, Brisbane, QLD 4059, Australia; g.moyle@qut.edu.au; 3 Counseling Services, Valparaiso University, Valparaiso, IN 46383, USA; stewart.cooper@valpo.edu

**Keywords:** anxiety, remediation, treatment, performance, sexual performance, stage performance, per-forming arts, sport psychology, performance psychology

## Abstract

Strategies for addressing anxiety-related decrements in performance have been implemented across a variety of domains, including Sex, Sport, and Stage. In this review, we (1) iterate the dominant anxiety-related remediation strategies within each of these domains; (2) identify over-lapping and domain-specific strategies; and (3) attempt to unify the conceptualization of performance-related anxiety across these three areas under the information-processing framework of the Reflective/deliberative—Impulsive/automatic Model (RIM). Despite both diversity and similarity in remediation approaches across domains, we found that many strategies appear to share the common goal of maintaining a dominant automatic style of information processing in high performance demand situations. We then describe how various remediation strategies might hypothetically fit within the RIM framework and its subcomponents, identifying each intervention as falling into one or more broad categories related to achieving and/or maintaining dominance in automatic information processing. We conclude by affirming the benefit of adopting a unifying information-processing framework for the conceptualization of performance-related anxiety, as a way of both guiding future cross- and inter- disciplinary research and elucidating effective remediation models that share common pathways/mechanisms to improved performance.

## 1. Introduction

Anxiety has long been associated with performance problems in a variety of fields and, not surprisingly, remediation often includes anxiety-reduction techniques such as cognitive reframing, relaxation, mindfulness, emotion management, and other coping strategies to address the problem [1,2,3,4,5]. Anxiety—defined as a negative mood typically accompanied by bodily symptoms such as increased heart rate, muscle tension, a sense of unease, and apprehension about the future [6]—can serve as a strong motivator for future avoidance and/or approach behaviors. 

Anxiety is tied to many types of performance, especially when an evaluative com-ponent is present [7,8]. Furthermore, such evaluation often entails significant consequences for an individual, which may further intensify the anxiety [9] (Our focus is on anxiety related to performance, competition, and winning, and not anxiety associated with organizational or personal issues). For example, partnered sexual activity, particularly at the onset of a relationship, typically includes both evaluative and consequence components, with sexual impairment having long been associated with anxiety [10,11,12]. In sport, athletes are often under scrutiny not just by teammates and coaches but also by fans, and the consequences for failed or diminished athletic performance can be both personal (e.g., embarrassment, loss of confidence, etc.) and professional (e.g., loss of opportunities, contracts, income, etc.) [13]. Stage performance shares similar characteristics. Whether it is a music, theatre, or dance performance, or even public speaking, each consists of evaluation by the audience (and critics), coupled with personal and professional consequences. Furthermore, when the performance involves collaborators, the success of the team, group, or partner can be adversely affected [14], with evaluation from partners or teammates having a profound effect [15].

Such anxiety undoubtedly occurs in other activities (e.g., academic test taking, work environments); however, situations involving Sex, Sport, and Stage all represent interpersonal performance domains (unlike test taking or math anxiety). Each has a strong self-evaluative component as well as an “other” evaluative component; and for each there may be significant consequences related to performance failure. These interpersonal aspects are known to provoke anxiety which in turn may affect performance and may eventually lead to avoidance. Examples of anxiety affecting performance have been well documented in all three fields (See Rowland and van Lankveld [16] for examples, or search online for “famous performers or celebs with stage fright” or “famous athletes with performance anxiety”).

At the same time, while anxieties related to Sex, Sport, and Stage share the common elements of evaluation and consequence, they also differ in substantive ways. For one, the response sets are different. For example, in Sex, autonomic responses related to sexual arousal are most salient; in Sport, visual/spatial motor responses may often dominate; and in Stage, cognitive/memory (including motor memory) processes are engaged, sometimes fully memorized, other times aided with musical scores or written notes. Second, Sport and Stage are carried out in highly competitive environments, from tryouts and auditions—where competition for coveted positions is intense—to public performances in either solo or team formats. Sex is less competitive, usually an intimate performance involving two people. Because they share similarities yet also differ in a number of respects, these domains provide a unique opportunity to examine the idea that performance anxiety related to different domains might be understood under a unified conceptual framework.

In a recent critical analysis of anxiety’s role in three areas of performance—Sex, Sport, and Stage—Rowland and van Lankveld [16] assessed the different ways in which anxiety has been approached and understood, identifying similarities and difference across the fields. That analysis revealed that although the evaluative nature and consequences of performance failure, as well as the varied motor performance response sets, differed across domains, striking similarities were also found. For example, all three fields recognized the relevance of specific situation variables (e.g., initial sexual encounter with a new partner, auditioning for a dance company, competing at the Olympics), specific task variables (e.g., drinking substantial alcohol prior to sex, singing a difficult aria, completing a decathlon) and specific subject variables (e.g., internalizers vs. externalizers) as risk factors that may increase anxiety and impair performance. Further, all three fields recognized that such factors did not function in isolation, but rather interacted at various levels to increase or decrease anxiety and thus to interfere with or facilitate response sets (e.g., a vulnerable disposition coupled with a particularly difficult task had a high probability of eliciting performance-interfering anxiety).

**Figure 1:**
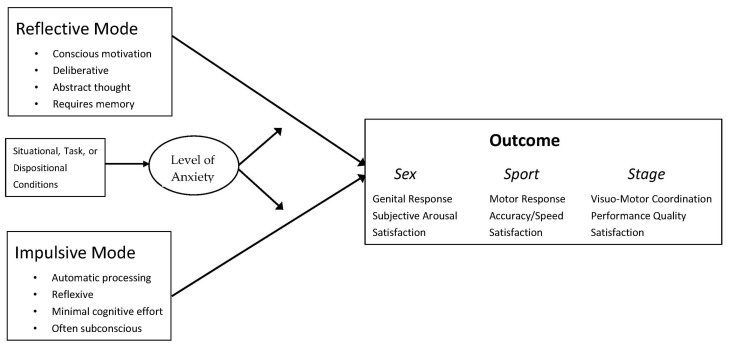
A reflective-impulsive model showing the association of moderators on functioning in the domains of Sex, Sport, and Stage performance.

Perhaps most striking, however, Sex, Sport, and Stage have each emphasized the importance of developing an automatic style of information processing. All three have investigated how anxiety, cognitive distortion, loss of attentional focus, and negative framing interfered with performance by shifting focus from automatic processing to a more deliberative/reflective style of processing. In response to this shared conceptualization, Rowland and van Lankveld [16] suggested that the understanding of performance-related anxiety might benefit from a unified conceptual model and common language that could enhance research, practice, and remediation across the three fields. Specifically, they proposed an adaptation of the reflective-impulsive model (RIM: [17,18,19]). This model, as applied to the dynamics of performance anxiety in Sex, Sport, and Stage (Figure 1), postulates that both *impulsive/reflexive* and *deliberative/reflective* information processing contribute to performance outcomes, but that the relative contribution of each mode is affected by various situational, task, or dispositional factors (affecting performance anxiety), which in turn can affect/alter performance.

### 1.1. Aims of the Current Review

As part of their review, Rowland and van Lankveld [16] established a roadmap for future directions, one of which was a comparison of the commonalities and divergences among intervention strategies, and hence the purpose of the present paper in which we focus on remediation/treatment strategies for overcoming performance-related anxiety in the fields of Sex, Sport, and Stage. Specifically, in this review we ask (1) what are the dominant remediation strategies within each domain; (2) which strategies overlap and which are unique; and (3) whether the RIM might serve as an overarching model for conceptualizing, unifying, applying, and developing effective remediation/treatments across the three domains. We conclude this final section with recommended future directions.

### 1.2. Methodology

A systematic review of the literature was carried out using relevant and multiple da-ta bases for each of the domains separately, using keywords related to anxiety, performance anxiety, performance (including domain specific terminology such as “choking” and “dysfunction”), cognitive processes, remediation, treatment, education, and so on. Results of these searchers were augmented by resources known to the authors through their expertise, as each author has had extensive experience in research, publication, and workshop presentations in the respective fields of Sex, Sport, and Stage.

## 2. Treatment/Remediation Strategies

Each of the fields—Sex, Sport, and Stage—attempts remediation using a variety of strategies. In this section, we discuss major approaches within each field. Here, we felt that that it was important that the readership understand that each domain has approached the problem of performance anxiety differently, often using different terminology, assumptions, and categories of inquiry. In order to be “true” to the discussions/research within each of the domains, we have adhered to the approaches and thinking within those domains.

### 2.1. Strategies Related to Remediating Sexual Anxiety

Treatment approaches related to sexual performance anxiety rest on principles first delineated by Masters and Johnson [20], Kaplan [21], and later, Barlow [4], and typically involve cognitive, mindfulness, emotion-focused, behavioral, and couples approaches. 

*Cognitive approaches* are concerned with perceptions, thoughts, feelings, and inter-pretations related to and during sexual activity. The underlying assumption is that anxiety, faulty beliefs, and unproductive/distorted thought processes can play a role in a person’s (or couple’s) experience of a sexual problem. Cognitive approaches focus less on the dysfunctional response itself and more on the client’s interpretive disposition toward the problem, with the goal of improving his/her self-efficacy and attitude [22,23,24]. Distorted thinking can intensify negative beliefs and emotions [25], leading to the expectation of “inevitable” failure and exacerbating the problem. As a result, an individual with performance issues may focus more on their fears and anxiety—and how to mitigate them—than on ongoing sexual stimulation. This anxiety can lead to “spectatoring,” referring to the attentional drift from ongoing sexual stimulation to monitoring and evaluating the adequacy of one’s own responses (e.g., am I getting or losing an erection or getting closer to orgasm) [26]. Cognitive approaches, then, encourage developing a positive internal dialogue that counters and prevails over self-defeating and unproductive beliefs, and that targets faulty coping mechanisms, giving clients a sense of control over their problem and improving their confidence and outlook regarding sexual activity [23,27]. 

*Mindfulness approaches* are closely linked to cognitive strategies but differ in their focus and objective. In contrast to the emphasis on countering particular thoughts, mind-sets, or emotions, mindfulness first encourages the development of a strong sense of self-awareness—an internal, passive state focused on the present and ongoing experience—then teaches clients to witness their thoughts, reactions, and emotions in a non-judgmental and accepting manner, to see them no differently than any other sensation or experience, that is, as something that arises and then subsides in one’s phenomenological experience. This approach builds on cognitive approaches, with the core tenet of “experiencing without passing judgement” applied to a sex therapy context [28]. Mindfulness sex therapy is particularly concerned with the bodily sensations and awareness of the five senses, in the present moment [29], including sensate focus [20], which helps individuals maximize their response by becoming more aware of their bodily sensations and by diminishing the performance demands of the situation. Mindfulness techniques have been quite effective, even when a sexual problem such as erectile dysfunction is somatic in origin. Of particular promise are techniques associated with mindfulness-based stress reduction (MBSR) [30] and mindfulness-based cognitive therapy [31], though to date they have seen only limited application to the treatment of sexual anxiety and performance.

*Emotion-focused approaches* use the negative emotions experienced from sexual dysfunction as useful sources of information, typically considering emotion as integral to information processing [32]. This therapeutic approach helps the client increase emotional awareness, regulate emotions, and replace negative emotions such as anxiety with positive ones. An emotion-focused approach assumes that while many emotions may be associated with negative sexual experiences, strong negative emotions from a failed sexual situation are likely to worsen the situation, overpower positive feelings about the situation, and leave the client feeling powerless [22]. As part of the therapeutic process, clients come to realize, first, that emotions are within their control and therefore can be directed more positively and thus productively, and second, that emotions surrounding a situation do not necessarily accurately reflect the outer world. In addition, when couples are involved in the process, they are coached to learn to accept that expectations of partners will not always align, nor will expectations always meet with the reality of a situation, similar to the concept of radical acceptance embodied in acceptance commitment therapy [33].

*Behavioral approaches* view sexual dysfunctions as a learned—likely conditioned—behavioral response to stimuli. Given that much of sexual response is physiological, a behavioral focus assumes an alternative approach that explores the manner in which sexual stimuli are presented, received, and responded to. This response can then be changed by conditioning the client to have more “appropriate” or desired responses through stimulus control. Behavioral approaches often focus on the complementary actions between the client and partner. Techniques include alternating stimulation and withdrawal, a focus on partner arousal or needs, specific intercourse positions and maneuvers, and masturbatory exercises, often supplemented by varied movement patterns, muscle relaxation, deep breathing, and movement with less muscular tension [23,34,35,36,37]. 

Behavioral techniques have been used with some success with men with premature and inhibited orgasm, and with women having arousal, orgasmic, or pain problems [38,39]. For example, men and women can focus on appropriate stimulation to adjust their responses during a partnered experience, using specific behaviors that either speed up or slow down the client’s and partner’s arousal levels [23]. Behavioral techniques have the added benefit of reducing blame/shame in the client, as the focus is on modifying behavior through the use of more appropriate stimuli, rather than changing the individual him- or herself. 

*Couple’s (relationship) approaches* to the remediation of sexual problems connect the performer directly with the “evaluator,” with a major goal of having a joint effort in the exploration and treatment of the problem [40,41]. The main tenet of a couples-focused treatment is that regardless of which partner is experiencing the problem, the couple as a whole is affected, as the sexual relationship is impaired. This attitude effectively negates the perceived “evaluator” role of the partner. At the core of couple-focused therapy is open communication and shared responsibility. This approach—which has been used effectively for both male and female sexual dysfunctions—removes the sting of shame, the fear of disappointed expectations, and the confusion of miscommunication and assumptions. 

Couples therapy can also address issues outside the sexual dysfunction that might impede progress, for example, erection problems that result from the negative interactional style of the couple, or low desire that may reflect the emotional distance of a lagging relationship rather than a specific sexual issue. In addition, the couple can “externalize” (de-personalize) their sexual challenges by forming a team that focuses on both improving their sexual performance and satisfaction as well as their overall relationship. Couples therapy for sexual problems focuses on “optimization” (as opposed to “pathology,”), emphasizing communication [42], behavioral change [43], and solutions [44,45], which can be used to leverage multiple dimensions of the relationship to ameliorate the sexual problem.

#### Section Summary

Table 1, Column 2, summarizes the behavioral, cognitive, mindfulness, emotional, or interpersonal interventions nested under Sex. Combining more than one technique is the norm [46], as is tailoring interventions based on the client’s individual characteristics and problem [47].

### 2.2. Strategies Related to “Choking” and Competitive Sport Anxiety 

Some of the first documented uses of psychological interventions in Sport were introduced by Coleman Griffith who conducted research and consultation with athletes and coaches at the University of Illinois in the 1920s and 30s [48]. Other pioneers in the field included Ogilvie and Tutko who published their seminal text *Problem Athletes and How to Handle Them* in 1966 [49], and Greenspan and Felz’s [50] review of research studies into the efficacy of sport psychology interventions with athletes. 

Nowadays, sport psychology offers several approaches for the treatment of competitive sports anxiety and choking (i.e., decreased skill execution and performance resulting from increased anxiety under perceived pressure) [51]. Some approaches are theoretically driven, while others are supported empirically. Given the extensive research emphasizing the roles of elevated anxiety and ineffective attentional processes [52], Sport interventions related to choking and competitive sport anxiety are discussed together. 

*Cognitive-behavioral approaches* are often combined to address specific situations in Sport (e.g., performance under pressure), and therefore clear distinctions between the two are not typically drawn. From a cognitive perspective, reframing experiences, in-creasing positive self-talk, engaging in cognitive restructuring, and moderating expectations may decrease the likelihood of choking [52,53]. Behavioral interventions such as pre-shot routines, relaxation techniques, and quiet eye training have also shown effectiveness.

Adapting to pressure and effectively managing anxiety lessens the impact and occurrence of choking [54,55,56]. Furthermore, conditioning athletes to perform under pressure in both simulated and actual situations is a commonly used strategy, along with the use of graded pressure situations during practice [56,57,58,59,60]. Additionally, trust monitoring—developing confidence in one’s athletic routine—can mitigate the debilitating effects of pressure. 

Building confidence through *productive thinking* (i.e., reframing unproductive self-talk with productive self-talk) increases athletes’ self-belief and therefore their ability to cope with the performance demands at hand [61]. When athletes realize that pressure (i.e., stress and task demand perceptions) is often self-generated, and further that some of this pressure is within their control (i.e., controlling the controllables), they can focus more on performance quality and less on the distracting aspects of the pressure. Additionally, athletes may be advised to focus on the normality of making mistakes, which then mitigates the effects of the pressure they might be experiencing or self-generating [62].

*Preparation*, which is similar to conditioning for performing under pressure, is a focal area for all athletes in both training and competition scenarios. Preparation includes both mental/cognitive and physical factors, the latter involving body-to-mind and mind-to-body approaches [13,53]. Physical preparation may include controlled breathing, engaging in progressive muscle relaxation (PMR) to recognize and release bodily tension, and using visual and auditory biofeedback to help athlete’s learn to regulate arousal and stress/anxiety responses [53]. The inverse—mind-to-body strategies such as autogenic training, meditation, and mindfulness—focuses on the use of imagery that induces physical relaxation. Based on individual differences, athletes usually incorporate one or more of these physical strategies to complement mental preparation techniques.

Examples of mental preparation include relaxation exercises, thoughts and feelings of pressure management, pressure acceptance, and recalling reasons for playing the game [63]. Additionally, *pre-performance routines* (PPR)—the use of performance related thoughts and behaviors prior to performance—have been used as an immediate preparation technique to prevent choking [53,57,59,62,64]. The use of PPR can enhance focus, manage anxiety, and increase self-confidence and perceived control [59]. An example of PPR is thinking of the word “relax” simultaneously with physical relaxation [62] and/or using imagery of the target tied to various positive scenarios [57].

*Coaching-led approaches* assess an athlete’s *focus style* (e.g., whether the focus is on the process of the movement rather than the goal/outcome of the movement). In doing so, a coach can intervene prior to performance problems and correct the athlete’s focus style [65]. Furthermore, the mastery approach to coaching (MAC) encourages coaches to use positive reinforcement, positive correction, and encouragement to overcome mistakes. MAC also emphasizes effort over outcome, which allows for personalized goal development for athletes as well as the promotion of enjoyment as an important part of their athletic activity [66].

Other coaching-led interventions flow directly from conceptual models of choking. For example, explicit monitoring theory (EMT) interventions use dual-task training in which players perform a task while attending to extraneous stimuli [67]. While this training appears to contradict the theoretical basis for attentional control theory (ACT), small amounts of distraction (e.g., listening to music) may induce a mild mental pre-occupation that helps athletes de-focus on explicit movements, which counter the more desired automatic processing mode [64]. Similar to EMT focused interventions, ACT based treatments have been implemented with some success by having players focus on the simple tasks at hand instead of complex goals [63]. To keep these tasks simple, holistic cue words are suggested such as “smooth” or “easy” in order to block focusing on explicit physical movements [68].

*Emotion-focused approaches*, based on the close tie between anxiety and choking, use cognitive and other approaches to manage negative emotions. Specifically, negative self-talk stimulates negative emotions, adverse outcomes, and self-defeating strategies [69], and when combined with negative instructions (“don’t miss”), the resulting anxiety can lead to choking [5]. Conversely, a facilitative interpretation of anxiety, also often impacted by self-talk, can be associated with better performance outcomes [70,71]. To counter negative self-talk, cognitive restructuring—the process of identifying and challenging maladaptive thoughts with positive self-talk—can help alleviate the anxiety and negative affect and reliably leads to lower likelihood of choking [57]. Even after a negative experience, positive self-talk, including thoughts related to success and positive interpretation of mistakes [72], can have beneficial effects. Combining such cognitive restructuring with relaxation exercises can provide an even more powerful strategy [59].

Performance-interfering negative emotions and anxiety can also be mitigated through other means. For example, athletes are encouraged to examine their performance (including errors) in segments, so as to avoid feeling overwhelmed by an endpoint of failure. In doing so, they can learn incrementally from each segment and attempt to either replicate or change their next performance [63]. Dealing with anxiety, however, extends beyond just countering the negative; it replaces negative reactions to a bad experience with more productive positive emotions related to resilience, control, and concentration. Such positive emotions can be elicited by many methods, including listening to music, viewing a humorous film, or recalling previous successes [73]. Athletes are further encouraged to view their negative experiences with objectivity in order to use them as learning experiences and to de-emphasize any associated negative feelings [63].

*Behavioral approaches* such as quiet eye training (QE) [74,75] involve the use of visual control by fixating on a relevant target just prior to performance. For example, athletes are trained to fixate on the outcome (goal) and then the object of the goal (ball), with the gaze remaining on the object (e.g., the ball, rather than arms, club, or other irrelevant objects) through and after the execution of the motor performance. QE-trained athletes have better visual attention control, are more accurate, and better maintain high levels of performance under pressure situations, all of which decrease the likelihood of choking [76].

#### Section Summary

Table 1, Column 3, summarizes interventions commonly used in Sport. The reader will note that techniques described under the Sex section also appear here since many of the cognitive and mindfulness interventions are routinely employed in both Sex and Sport [61,77]. At the same time, several remediation strategies are unique to Sport, for example, developing productive thinking to mitigate performance loss under intense pressure, quiet eye training, and pre-performance routines [53,57,59,62,64].

### 2.3. Strategies Related to Stage Anxiety 

While a focus on treatment of the pathological aspects of this unique population (e.g., anxiety, depression, eating disorders) developed over time [78,79], the specific application of Sport strategies to the performing arts was only seriously considered towards the end of the 20th Century [80,81,82,83]. Drawing from sport psychology, performance psychology has since evolved into its own subfield, with research identifying many parallels to, and similarities with, performing arts domains [84,85,86,87,88]. 

Anxiety within this context is usually referred to as “performance anxiety” as it manifests in situations where a performative element occurs, such as dance, music, acting, or public speaking situations. However, here we refer to it as “stage-related anxiety” to distinguish it from sexual performance anxiety or a more generalized anxiety related to any type of performance (e.g., test-taking). Similar to Sport, excessive stage anxiety can interfere with the mental and physical execution (i.e., performance) of skilled movement, particularly when the performer must demonstrate a high level of skill under close scrutiny by an audience, in comparison or competition with others (hence, evaluation) [89]. 

The effective management of stage-related anxiety has included cognitive, behav-ioral, and affective approaches common to Sex and Sport: reframing; positive self-talk; problem-focused coping; imagery; relaxation, breathing and mindfulness; and increasing positive self-efficacy and self-esteem [90,91,92,93,94]. Furthermore, coping mechanisms relying on social support and interaction (e.g., joking) also appear helpful [88,95]. 

#### 2.3.1. Specific Organizing Strategies

Performing artists are often advised that the risk of performance anxiety can be re-duced by attending to a number of personal, task, and situational factors. 

*Personal strategies* refer to the individualized strategies performers use to manage or decrease anxiety as they cope with the challenges of performance. Such strategies—observed among youth as early as 11 or 12 years [94]—entail the development of idiosyncratic responses, whereby performers try a variety of tactics to manage the causes and effects of anxiety as they develop an individualized “tool kit.” Some such problem-focused coping mechanisms (e.g., planning, seeking social support for instrumental reasons) may be effective; however, others—including some emotion-focused strategies (e.g., wishful thinking, self-blame, venting of emotions)—may be counterproductive or maladaptive [90]. Ultimately, performers need to develop individualized strategies that boost confidence in their ability, develop effective self-evaluation skills for correcting mistakes, and emphasize self-care, as strong performance requires mental, emotional, and physical vitality [91].

*Task-oriented strategies* are similar among the various performing arts domains. For example, musicians are encouraged to select material carefully (i.e., when they can do so) so as to achieve enhanced performance by learning the piece deeply, managing practice/rehearsal time closely, experimenting with the best way to perform the material, and developing spontaneity in its delivery. Practice needs to be corrective and productive, such as emphasizing the conditions precipitating an error rather than the error itself. More specifically, countering anxious habits with foundational knowledge and skills can prevent anxiety, as continuing to practice the same skill set without resolving the error only perpetuates the problem. Extensive practice and training [96] help performers learn how to be “in the moment” (i.e., a mindfulness approach), thinking only about the music—such as crescendo or decrescendo rather than finger placement or the amount of air (for vocalists)—and enabling refocus even in the face of major distraction from the audience (e.g., picture taking, cell phone ringing [97]). In dance, overlearning and subsequent automaticity is used to cope with a potential source of anxiety, such as forgetting the choreography, and to enhance the emotional communication of the work [95]. 

*Situational strategies* involve “controlling the controllables,” such as becoming accustomed to the performance setting, developing presentation skills, adopting skills that maximize performance when performing with others, and being organized [61,91]. As performers must demonstrate their skills in many unfamiliar settings, a key part of preparation includes familiarity with the performance setting (e.g., lights, stage set-up, props, and audience) so they are unperturbed by spontaneous situations and unexpected distractions. To best address this, performers typically practice their entire performance multiple times as part of a standard rehearsal schedule, culminating in several “dress rehearsals” designed to emulate the actual performance [91,98]. Furthermore, planning out practice/rehearsal time and establishing pre-performance routines (PPR) minimizes pre-performance anxiety, as such general performance management skills help performers stay centered, calm, and controlled during situational stressors [99]. 

#### 2.3.2. Cognitive-Behavioral Approaches

Beyond the three organizational categories above, effective management of stage performance anxiety has used a variety of strategies similar to both Sex and Sport contexts. 

*Cognitive approaches*, including cognitive restructuring, are designed to change faulty thought processes that create maladaptive behaviors such as avoidance and impaired performance. They also attempt to replace negative and unproductive thoughts with more rational and effective thoughts to gain insight into the problematic situation, making the anxiety-producing situations more controllable [100]. Cognitive restructuring may also involve self-talk or narrative, as self-talk can help performers modify their view of risk. For example, rather than trying to block out the audience, the situation might be reframed such that connection with the audience is viewed as a performance enhancer [17]. 

A cognitive-behavioral strategy sometimes used in acting, called the *confidence stimulus* or *confident character*, has demonstrated efficacy as well [101]. This approach relies on the acting technique of using personal experiences and stimuli to generate emotional states of characters, for example, thinking about a personal loss in order to express grief. This training first involves identifying and assessing appropriate confidence stimuli, then learning disciplined concentration, and finally practicing the art of conjuring up the confidence stimulus when under peak duress, fear, anxiety, obstacles, and distraction [101]. As part of the process, performers learn to actively switch thoughts from anxiety-provoking topics to their confidence stimulus. 

*Behavioral approaches* are aimed at changing problematic behaviors that arise from stage-related anxiety and include the use of deep progressive muscle relaxation (PMR). Such techniques reduce tension that causes motor movement patterns that interfere with smooth, coordinated performance, allowing movement to become more fluid and furthering deliberate and conscious control over posture and movement [53]. Furthermore, using kinesthetic cues (i.e., the sensations of tension, effort, weight, and position in space) to organize one’s field of awareness in a systematic way is an important element of feeling in control [100]. Systematic desensitization is also used to reduce panic prior to an actual speaking or stage event. This process encourages the use of guided, relaxing imagery while engaging in fear/anxiety-provoking situations in gradual steps, until the performer can visualize the situation without any muscle tension [102,103]. Once the fear has been overcome, the performer uses the learned tactic in actual performance situations, with the reactive response to the anxiety-inducing stimuli effectively dulled [100,104].

*Mindfulness approaches* have been used extensively to assist in the management of stage-related anxiety. For example, music students participating in yoga programs show decreases in both somatic and cognitive anxiety, reducing their shaking, racing heartrate, fear of negative evaluation, and fear of failing [105]. Compared to non-participants, they also experience larger decreases in both state and trait anxiety measures [105] (It is not clear precisely how trait anxiety, presumably an enduring dispositional characteristic, might have been affected by the yoga training). Meditation-based approaches similar to mindfulness-based stress reduction (MBSR) and mindfulness-based cognitive therapy (MBCT) have assisted singers and dancers in decreasing stress, increasing general relaxation and focus before performing, and enhancing performance and artistic creativity (see [106]). 

#### 2.3.3. Group Approaches

Minimizing the stigma associated with stage-related anxiety can often be achieved through group discussions and disclosure, which can help normalize the condition [17]. Such group-focused interventions may be further reinforced by directing learners to the numerous sources dedicated to helping performers counter stage-related anxiety, including websites, books, and blogs [98]. Such sources often emphasize the near universal nature of performance-related anxiety and the many strategies used by colleagues to counter it. Finally, given that stage performance often involves multiple people and teams, development of productive colleague interaction and group-based anxiety reduction strategies are common.

#### 2.3.4. Section Summary

As noted in Table 1, Column 4, a number of the behavioral, cognitive, emotional, and mindfulness interventions are utilized in Stage, and some of these are common to remediation in Sex and Sport as well. At the same time, several unique strategies are used in this field, primarily related to moving practice toward the successive context of the actual performance, (e.g., dress rehearsals).

### 2.4. A Summary of Commonalities and Contrasts

The commonalities and contrast among interventions to reduce performance-inhibiting anxiety in Sex, Sport, and Stage can be imaged as a Venn diagram. At the common center are use of cognitive, emotion focused, mindfulness, and life-style/life-perspective approaches. Examples of these would include instruction in the use of applied relaxation, self-talk, imagery, goal setting, and concentration techniques. The unique aspects of Sex, Sport, and Stage anxiety management are techniques which have emerged from the work of performance psychologists embedded, respectively, in each of these three domains. These domain-specific interventions are employed only in that particular field. An example from Sex is the stop-pause and partner stimulation method. An example from Sport is quiet eye training. Additionally, an example from Stage is use of multiple standard rehearsals leading to a formal dress rehearsal.

## 3. Intervention and Remediation: Integration

### 3.1. Overview of the Structure of Interventions

One of the challenges of taking a cross-disciplinary approach is that of finding com-mon constructs that underlie the different avenues of inquiry, language, and terminology across domains. In this section, we attempt to identify common elements and categories that enable more direct comparisons, and thus integration, across the three domains of Sex, Sport, and Stage. In some instances, this was readily apparent but in others less so. Nevertheless, the shared outcome achieved through implementation of any and all of the outlined remediation or intervention strategies is to improve performance by effectively managing anxiety. 

Structurally, interventions across all three domains may be placed into common change focus areas: awareness, cognition, behaviors, interactions, biology, and environment. In this section, we first note the way in which remediation/intervention is typically implemented across disciplines. We then iterate remediation strategies that (1) appear to be primary and common to all three fields, (2) are related, but are secondary or somewhat different across the three fields, or (3) appear to be primarily domain-specific. 

### 3.2. Intervention Delivery Formats

Some differences across domains are tied to the primary unit/object of the intervention. For example, interventions can be targeted towards, and delivered across, multiple levels, including the individual, couples, team/group, and systems/organizational. In Sex, remediation most typically occurs in a one-on-one or couples’ format. Less frequently, interventions are delivered in a group-therapy format, the latter providing social support from peers and a sense of diminished stigma. In Sport and Stage, interventions are delivered through a variety of formats including individual, team/group, sports program/performance company, and organizational-wide. Furthermore, the interventions can be delivered concurrently across multiple stake-holder groups, and can include other people such as: (for Sport) coaches, support staff, program managers, executive management; and (for Stage) artistic staff (e.g., ballet master/mistress, rehearsal director, choreographer, teacher, musicians), administrative staff, and executive management. Group level interventions are often preferred when working with a team sport, including the use of the couple’s intervention format for duos working as a team, whether or not intimately involved. Within Sport contexts, significant importance is given to addressing organizational and team dynamic issues via interventions targeted across these levels. Specifically, positive interpersonal relationships, a helpful team culture, and effective organizational operations and communication are deemed essential for supporting desired performance outcomes at both the team and individual level [107,108]. Although many Stage settings are similar, an understanding of the specific performing arts discipline context is critical for effective interventions across individual, group, and organizational levels [87,98,109]. Many interventions are taught and practiced in environments simulating the actual performance setting, and the nature of the relationship between the intervention service provider and the performer may look quite different than other forms of help-providing. Further, interventions may be delivered in-person and/or via remote technology, the latter because the service provider and performer may not share the same location.

### 3.3. Examples of Core Strategies Common to the Domains

Although the language, terminology, response sets, and specific procedures may differ, considerable overlap exists in the remediation strategies across the three do-mains. In this section, we discuss the aims and procedures for various core strategies (Table 1).

*Cognitive approaches* tend to dominate the psychological approaches toward mitigating the effects of performance anxiety. Each domain, for example, promotes the value of cognitive reframing, and each provides specific ways to accomplish this task. Specifically, identifying faulty thinking and distorted beliefs is a key initial step in the cognitive approach. Once identified, a subsequent goal is to replace the faulty beliefs and assumptions with more objective and accurate assessments. As part of this re-structuring, clients develop and engage in positive thinking, positive self-talk, and narratives about themselves, with the goal of improving their sense of self-efficacy and esteem, and reinforcing a “can do” attitude. Such strategies help prevent or break the cycle of anxiety that often develops in situations where clients feel helpless or sense a lack of control/self-efficacy.

*Emotion-focused approaches*. Cognitive restructuring and managing anxiety are often implemented concurrently. As a classic stress management coping strategy [110], emotion focused approaches are often used when individuals feel unable to alter the situation or conditions that produce the stress and anxiety; in response, they turn to strategies that help mitigate the anxiety caused by the situation or event. By controlling anxiety, performers free themselves from its debilitating effects (e.g., interference with erectile response in Sex, diminished bodily control in Sport and Stage) known to interfere with optimal performance, whether the response involves autonomic/smooth muscle response (e.g., sexual response), visual-motor coordination (e.g., Sport and Stage), vocal/respiratory response (Stage), and so on.

Although each domain may achieve the outcome using different steps, the process typically involves a series of gradations that initially increase awareness of emotional states and their effects, then uses strategies to regulate emotional impact, and finally channels the emotional energy into more productive positive emotional responses that replace the negative feelings. These steps may be accompanied by re-framing the anxiety as a facilitative process that can enhance performance (the idea that moderate anxiety can be a motivating/productive drive). In both Sport and Stage, the strategy of training and adapting to performance under pressure may also be included, with successful performance during practice leading to greater self-confidence and efficacy. No matter the scenario, emotional regulation is a way of managing/mitigating anxiety while energizing more productive responding.

*Mindfulness approaches*. A third cross-domain strategy related to both cognitive and emotion-focused approaches utilizes mental/mindfulness techniques. Such techniques help keep the performer “in the moment,” attending to and deeply concentrating on the stimuli relevant to accomplishing the task at hand and muting distractive thoughts related to both ongoing situational factors and consequences of errors/failure. In focusing on the present reality, especially sensations and ongoing experiences, mindfulness emphasizes a non-judgmental perspective that diminishes a sense of demand and counteracts response-monitoring (e.g., spectatoring) and its negative effects, as is seen with *sensate focus*, a longstanding strategy used for individuals suffering from performance anxiety surrounding sexual response. Several adjunctive techniques may be used to achieve a “mindfulness” state, including mental imagery, relaxation, controlled breathing exercises, meditation/yoga, and quiet eye training. The use of mindfulness thus relies heavily on an automatic processing of information as opposed to setting a deliberate course of thinking that projects into the future, and it does so in an emotionally attenuated manner—thus drawing on both the cognitive and emotional systems within the individual. 

*General lifestyle and life-perspective approaches*. In addition to cognitive/emotion strategies that include self-talk, mindfulness, relaxation, achievable goals, and concentration, other common approaches can be readily applied to Sex, Sport, and Stage. For example, Kiser [111] identifies the importance of “energy management” through consistent sleep patterns, a healthy diet, exercise, restorative intervals of downtime be-tween periods of intense physical, and mental activity that helps maintain the cognitive resources needed to self-regulate. Another strategy—common to all three domains—is that of expanding one’s sense of identity beyond the activities and actions related to the performance under question. For example, individuals can put their performance in better perspective by finding a broader basis for self-definition, one that includes elements beyond those of the individual’s professional (or sexual) identity/life.

### 3.4. Examples of Related/Secondary Strategies

Several remediation strategies are given greater emphasis depending on the specific domain. Yet, they may share common elements across domains (Table 1). 

*Couples/team approaches*. Within the Sex domain, the couple is often viewed as the target unit; however, duos are not uncommon in both Sport and Stage. Whereas Sex involves a personal/intimate relationship, Sport and Stage more often represent a professional relationship, for example in figure-skating pairs and competitive/artistic dance (e.g., Latin Dance, Ballet pas de deux). No matter the situation, because the performance of one partner impacts the overall performance of the duo, with each partner thus being evaluated by the other, taking a couple’s approach can provide value for performing duos across all three domains, even when intimacy is not involved. 

Broadly speaking, couples’ therapy emphasizes both the shared nature of the problem and a collaborative approach to its resolution. Performance issues can thus be “externalized,” with collaboration taking precedence over “evaluation.” The communication- [42], behavioral- [43], and solution- focused approaches of couples therapy include remediation principles for addressing competitive/stage-related anxiety and performance issues that could readily apply to both Sport and Stage [44], but which are perhaps less commonly implemented due to the mistaken assumption that they apply only for romantically involved couples. Couples therapy can also play an important role when performing duos in Sport and Stage are romantically involved, as issues within their personal relationships can negatively impact their professional interactions and performance. In addition, a strong professional relationship within a duo might elicit issues with an external romantic partner (i.e., not involved in the sport/performing art) which could also affect performance [112]. In such scenarios, couples therapy might explore solutions to personal issues, while simultaneously addressing potential effects on professional performance. In essence, the couples approach helps lessen the evaluative component of performance while concomitantly in-creasing the social support component.

In many respects, the couples approach to sex therapy embeds a number of strat-egies used in the team approach for Sport and Stage. A key component of developing high performing teams (i.e., Sport) or an ensemble/corps (i.e., Stage) is establishing a team culture of “helping others” [113,114]. Strategies that focus on supporting effective team/group dynamics as part of a “helping” culture may include the establishment of peer leaders [115] and place emphasis on effective communication, shared problem-solving, contingency planning, shared goal setting, and values-based activities (i.e., team ‘frames’ and charters) [95]. 

*Personality dispositions*. Each domain has identified specific personality traits and/or dispositions that increase vulnerability to performance anxiety [16]. For Sex and Stage, these characteristics focus on negative affectivity, trait anxiety, introversion, and social phobia. For Sport, anxiety is seen as the dominant personality characteristic related to disrupted performance. All three domains also identify dispositional factors that increase individual vulnerability, such as low self-confidence, overly high expectations and perfectionism, and high self-consciousness; all are presumably characteristics that lower the threshold for triggering state anxiety. These personality dispositions are often the target of change in cognitive/emotional therapies discussed previously. In contrast, personality traits—presumed to be enduring characteristics resistant to change—have generally not been targeted by remediation procedures. 

*Physiological/physical/medication approaches.* Under this far-reaching category, various domains take quite different approaches, not surprising in part because the motor response demands differ across areas. However, the use of drugs/medication in all three fields has engendered debate and controversy.

In Sex, medications that either increase erectile capacity or delay ejaculation are sometimes used in conjunction with therapy, or simply on their own. Such drugs assist in enhancing a more appropriate sexual response. In doing so, they boost the man’s confidence and self-efficacy, ease the fear and embarrassment of failure, and thus reduce anxiety. Medications have been less effective in managing sexual issues in women and have been tied more to sexual interest than sexual performance. 

In Stage, medications such as beta blockers attenuate sympathetic nervous system activity, thereby relieving acute anxiety symptoms related to performance anxiety. In blocking the physiological manifestations of anxiety such as increased heart rate, stomach upset, and tremors [91], they eliminate the somatic triggers that initiate or intensify anxiety. The end result is greater control over critical bodily responses necessary for quality performance (e.g., fine motor performance, controlled breathing, and so on). For both Sex and Stage, medication represents a symptomatic approach that does not address the underlying problem. 

The use of medication in Sport is generally not considered a remediation procedure. Medication use remains controversial and, in many instances, is banned or counterproductive. Specifically, the use of drugs that offer competitive advantages are typically prohibited in most athletic competitions, and drugs that calm nerves may interfere with cognitive function and/or fine motor performance.

### 3.5. Examples of Domain-Specific Strategies

In addition to remediation strategies common to all three domains, each domain can make use of strategies that others cannot (Table 1). Sometimes these strategies are related to differences in the activity itself: for example, the specific response demands (autonomic vs. striate muscle), the setting/situation (e.g., private vs. public), the evaluative process and consequences (personal and/or professional), and the values/constraints of the discipline itself (e.g., trying to establish a level playing field vs. encouraging any type of enhanced individual performance). 

*Preparation and Practice*. To achieve a high level of automatic processing, Sport and Stage use practice and preparation. Specifically, high levels of proficiency result in a performance that becomes automatic—in some instances “rote”—and when combined with pre-performance routines, these steps ensure a higher level of self-efficacy and confidence. Furthermore, this automated performance may be subjected to increasing levels of pressure, both simulated and real, to help performers condition themselves to situations of increasing anxiety. Sex has no clear analog for “practice and training,” although other steps may be used to assist in the automatic processing of sexual stimuli. For example, mindfulness and sensate focus techniques, as noted earlier, shift attention away from the endpoints of erection/arousal and orgasm (as well as the potential failure to reach these endpoints) and toward the more relevant sensory information of the moment. In addition, Sex does include a number of behavioral techniques that can assist individuals “practice” on their own so that they can achieve greater arousal during partnered sex; such techniques might include genital exploration and stimulation for women who are anorgasmic, and the stop-pause and partner-simulated stimulation for men who experience premature or inhibited ejaculation.

Within the domains of Sport and Stage, one strategy that supports effective prep-aration and practice is referred to as “controlling the controllables.” Based on approaches that minimize uncertainty [88] through thorough preparation for “what if” scenarios [61], this strategy emphasizes an understanding of what can or cannot be done to control the situation (i.e., locus of control). Specifically, “controlling the controllables” requires athletes and performing artists to identify the factors they can control: mental preparation, physical training, practice, nutrition, rest, recovery, familiarity with their performance environment, and planning for unexpected events. Conversely, recognizing and accepting the factors they cannot control (e.g., weather, technical malfunctions on stage, officials’ rulings during a competition), and equally important, recognizing that they can still control their response to these uncontrollable factors, can strongly impact their ability to effectively manage their competitive or stage-related anxiety. 

*Domain-Specific Behavioral Strategies*. Sport and Stage can also make use of positive feedback, reinforcement, re-conditioning, and correction from coaches and instructors when errors are imminent or committed. Such feedback is intended to prevent negative framing of the situation (i.e., the instructor’s positive feedback is meant to overcome negative self-feedback), to encourage and motivate the performer, and to provide a behavioral cue to “reprogram” correct movement execution on subsequent attempts and assist with error recovery. Although not exactly parallel, in Sex, the partner is in the position to provide feedback, and if done sympathetically and in a non-demanding (but not dismissive) way, this type of communication can achieve positive results.

## 4. Relationship of Treatment Strategies to the Reflective-Impulsive Model

In this final section, we explore whether the Reflective-Impulsive Model (RIM) might provide an overarching framework for research on and treatment of anxiety-based performance issues across the domains of Sex, Sport, and Stage, with the central premise that a productive psychological model assists in (a) understanding, (b) prediction, and (c) control [116]. We then hypothesize ways in which existing remediation strategies might be subsumed under RIM, and, finally, we offer several recommendations for further research.

### 4.1. RIM: A Better Model to Meet the Goals of Understanding, Prediction, and Control

The long-time dominant model of the relationship between anxiety, arousal, and performance, known as the Yerkes-Dodson Law (YDL; [117,118]), postulates that, as stimulus strength rises, habit formation improves, but only up to a certain maximum, when it begins to deteriorate as stimulus strength continues to increase. This pattern generates the well-known inverted U-shape function. Task difficulty moderates this relationship: the optimal anxiety-provoking stimulus strength is higher for easier than difficult tasks. The Yerkes-Dodson model was intuitively appealing and gained much popularity over the decades, with the inverted U-function used to explain performance across a large number of settings, ranging from business, educational/academic, sport, and so on. Indeed, in support of this idea, moderate levels of anxiety do appear to enhance performance under a variety of conditions and activities. For example, moderate anxiety induced through situational pressure can improve erectile response in men who have no issues with dysfunction [119]. In a similar manner, Otten [120] has proposed an effect opposite to performance anxiety/choking termed “clutch performance,” defined as enhanced performance in the face of pressure. Additionally, with regard to Stage performance, a moderate amount of anxiety assists performance when the anxiety is reframed as performance energy or interpreted positively [97]. Nevertheless, the Yerkes-Dodson Law encountered criticism due to its lack of detail and its overly general applicability to the myriad of situations and performance types to which it had been applied (see [121,122]). 

In our view, a better model for exploring anxiety’s impact on performance in Sex, Sport, and Stage is the Reflective-Impulsive Model (RIM: [16,18,123]). The RIM belongs to a larger “family” of dual-process models [124] that posit the involvement of two highly different, independent, and component cognitive processes: (1) an impulsive/automatic system mainly operating out of conscious awareness, and (2) a reflective/ deliberative process involving frontal cortical activities. Specifically, the impulsive system is permanently active and operates automatically as it processes incoming information from the entire perceptual field, requiring minimal cognitive resources. Many of its operations occur outside awareness, although some may oscillate between conscious and subconscious experience. In contrast, the reflective system involves abstract, conditional, and hypothetical reasoning. As this latter system requires holding several bits of information in working memory, it can handle only small amounts of information and is highly dependent on the availability of processing capacity in working memory. 

From the standpoint of performance issues, automatic processing involves a set of deeply seated learned stimulus-response reflexes which, when replaced by a more intentional or deliberative processing style, can lead to interruption of these “highly programmed” response sequences. As an example, a cyclist may effortlessly perform all necessary movements and adjustments while processing information from the traffic situation, the road surface condition/obstructions, presence of pedestrians and animals, etc., and yet may simultaneously be immersed in an abstract conversation with a fellow rider. However, when the situation becomes highly complex (e.g., approaching a busy roundabout having many cars, pedestrians, and other cyclists but no traffic signal), the cyclist interrupts the conversation to refocus attentional resources to respond to the situation. In doing so, the reflective system can interrupt or override reflexive processing, and thus exert inhibitory control and/or activate different response systems [124,125]. If the need for processing capacity is again reduced, the omnipresent reflexive system again assumes priority in the control of behavioral output [126]. In summary we believe that RIM offers a fairly sophisticated lens for deepening our understanding of the anxiety-mitigating strategies that have emerged in Sex, Sport, and Stage. In addition, the model suggests the development of more focused possibilities for future interventions. 

RIM, as related to Sex, Sport, and Stage, is depicted in Figure 1. Further, situational, task, and dispositional conditions can affect performance anxiety which, in turn, can affective cognitive processing mode. The model also shows several specific goals for Sex, Sport, and Stage performance enhancement interventions. As applied to the dynamics of performance anxiety in Sex, Sport, and Stage, RIM would postulate the contribution of both impulsive/reflexive and deliberative/reflective processing to performance outcomes, with performance anxiety disrupting or altering the information-processing channel (reflective vs. impulsive) that dominates at any given moment.

Regarding treatment strategies for performance-inhibiting levels of Sex, Sport, and Stage anxiety, the model serves both as a framework for deeper understanding of how the interventions function and as a source for new intervention approaches that have yet to be developed. We propose that examining existing interventions for anxiety-related decrements in performance within the RIM framework could provide a fruitful starting place for establishing common ground across domains. Not only would this process help establish the degree of utility of RIM for the understanding of anxiety-related performance issues, but it would also allow prediction of specific outcomes as well as identify presumed processes underlying those outcomes. In addition, conceptual matching of intervention strategies with RIM assumptions could provide a vehicle for grouping discipline-specific variables/constructs/language (as well as their assessment) under common/shared rubrics that might encourage better cross-disciplinary communication, research, and intervention processes, enabling more facile movement into the use of RIM as means of control over producing specific outcomes. Ultimately, the goal of enhanced performance within all three domains could be attained.

### 4.2. Hypothesized Connections between Intervention Strategies and RIM

A number of intervention approaches endorse the idea that automatic processing of information is important for peak performance, and that disruption of this processing mode may well lead to performance problems. However, to our knowledge, no initiatives have attempted to interpret the variety of intervention strategies used in Sex, Sport, and Stage within the framework of RIM. Furthermore, although many studies have recommended the use of particular performance-improving strategies in these fields, for the most part they have *not* related the findings to a larger set of interconnected processes that might be explained by an umbrella theory such as RIM. This section is thus devoted to drawing potential connections between seemingly un-related intervention strategies in an attempt to demonstrate their possible fit within the larger RIM. As these connections have typically not been established empirically, we view our discussion as hypothetical, yet one that could suggest possible directions for future empirical research that might transcend disciplinary boundaries. 

**Figure 2:**
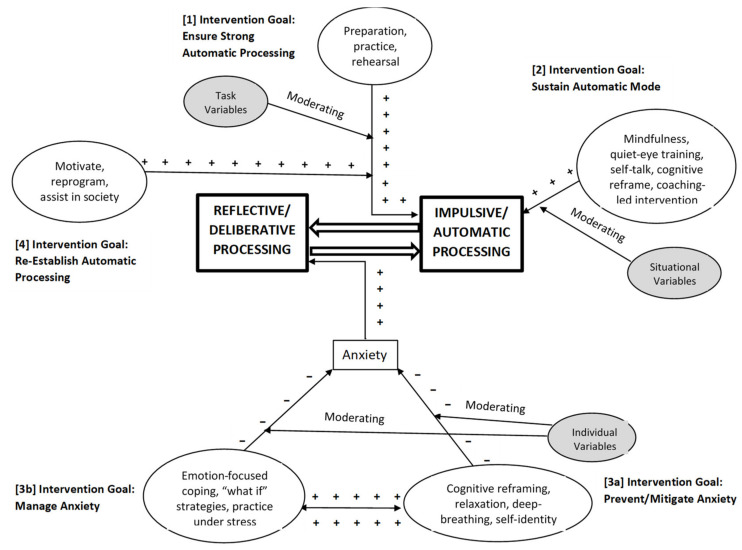
Scheme showing location of effect within RIM for the four intervention strategies (1,2,3a,3b,4) outlined in the text. Legend. (**a**) The two types of information processing are represented by the bold rectangles, with anxiety, represented by a smaller light rectangle, being a major source for retrogression to deliberative processing. (**b**) Intervention strategies (1, 2, 3a, 3b, and 4) are represented in bold font and corresponding ovals. (**c**) Moderating variables (task, situational, individual) are represented by the three shaded circles. (**d**) Signs (+ or −) represent facilitative/additive or inhibitory processes.

Perhaps in its most simplified form, interventions might be conceptualized as maintaining a balance between reflective and automatic/impulsive processing modes to ensure peak performance, with the assumption that performance is optimized by automatic-mode dominance during high demand Sex, Sport, and Stage performance (Figure 2). As such, interventions might be viewed as falling under one or more of four goals related to establishing and maintaining dominance of the automatic high-performance system. The first goal is to ensure that the performer has achieved transition from reflective processing mode to a proficient automatic processing mode. The second goal is to enhance the likelihood that the performer continues in (and is deeply engaged with) the automatic processing mode and does not revert mainly to reflective processing during performance. The third goal is to block or mitigate those stimuli/processes that have a strong potential, via increased anxiety, to revert the individual to the reflective mode. Additionally, the fourth goal is to re-establish, as rapidly as possible, the automatic processing mode in individuals who have, for whatever reason, lapsed toward or into reflective mode dominance during high performance activities. 

In this discussion we proceed from the more obvious/parsimonious connections between interventions and RIM to the less obvious/more complex and indirect connections. To review briefly, initial engagement in an activity requires a learning process involving substantial and deliberate reflective/verbal operations to generate appropriate responses [17,18,19]. That is, the learner rehearses verbally and spatially what action needs to be taken (e.g., hold the golf club in this position and swing with this body and arm movement) [16]. With practice and experience, response sets become increasingly automatic, reflexive, and non-reflective, not only requiring minimal forethought, but also becoming refined, accurate, and effective in the process. This pattern is particularly evident in viewing the results of the sustained deliberate rehearsal routinely practiced among elite performers [16]. In light of the above, the intervention strategy that most obviously helps (and perhaps is most long-standing) achieve this transformation is *preparation and practice*. Furthermore, the relevance of *task-related* variables (e.g., difficult vs. easy) is readily apparent to this transitional process.

A second broad remedial approach is to ensure that the individual continues to function in automatic processing mode when the situation demands high performance. The use of *mindfulness* (and its related process of *sensate focus*), *quiet eye training*, and *self-talk* represent various means of focusing attention on the here-and-now of automatic processing, preventing attentional “drift” toward deliberative processing (e.g., spectatoring, muting distraction) related to action (or mis-action) and its consequence, for example, when *situational* factors (e.g., high stakes) play an important role. 

A third set of interventional approaches are aimed at preventing or managing anxiety—the dominant experiential trigger most often responsible for retrogression to the reflective mode. These approaches may function by *preventing/inhibiting* the anxiety in the first place, by increasing thought processes that can help *neutralize/counter* developing anxiety (e.g., *cognitive reframing*), and by developing strategies (e.g., relaxation, deep breathing) to *mitigate/manage* the anxiety when it does occur. An underlying assumption inherent in this process is the recognition that emotion—in this case, anxiety—has long been conceptualized as an evolutionary mechanism that serves to quickly re-order priorities for the organism [127,128]. As such, performance anxiety disrupts automatic, uncompromised functioning in Sex, Sport, and Stage, shifting the performer’s focus to more deliberative, reflective operations. While deliberative operations are critical in the formative stages of automatic processing, they become an encumbrance during high level performance due to their demand for slower and more taxing information processing. 

Interventions related to this third category may include *relaxation, deep breathing, anti-anxiety medications, cognitive restructuring* and “*controlling the controllables*” (that enhance confidence and self-efficacy) and might be viewed as strategies designed to *inhibit/prevent* and/or *counter* anxiety. The relevance of personality dispositions to this process also becomes evident: for example, high anxiety types have substantially lower thresholds to and higher levels of “latent” or welling anxiety, and thus the trigger to disrupt automatic processing and reinstate the deliberative/reflective processing might be more sensitive in these individuals. 

Additionally, within this third category of interventional approaches is that of *managing the anxiety* that is likely to surface during high stakes situations. In many instances, an individual may be unsuccessful in inhibiting the negative emotional response, so some remediation processes are aimed at managing the anxiety that does occur, thus keeping the performer from retrogressing to deliberative processing mode. In addition to already-noted strategies used to counter anxiety (e.g., relaxation, deep breathing), *emotion-focused therapy*, *coping* [including “*what if*”] *strategies,* and *practicing under stress* might be included among such strategies. *Cognitive reframing* that emphasizes expanding one’s *self-identity beyond that tied to the activities related to the performance* under question offers a way of diminishing the perceived consequences of negative outcomes, thereby reducing the potential for debilitating anxiety.

Finally, a fourth set of interventions are aimed at helping to rapidly reinstate dominance of automatic processing in situations/individuals when retrogression to the reflective/deliberative mode has occurred. These strategies, though often not well de-fined, may include positive feedback and coaching (from a partner as well as a coach) designed to *motivate*, *re-program*, and *assist in recovery*, as well as additional *preparation* and *practice* designed to strengthen and further solidify the automatic processing mode. 

Evident from the above discussion, intervention strategies categorized by process (e.g., cognitive, behavioral, physiological, etc.) may use a variety of techniques to maintain dominance of automatic processing during performance, and therefore they may target multiple processes. For example, drug-induced states may affect different processes within the model: anti-anxiety medications and alpha blockers inhibit the rise of anxiety, whereas performance enhancers improve the likelihood of strong motor performance despite moderate levels of stress/anxiety. In a similar manner, both couple and team approaches incorporate a variety of techniques aimed at different steps/goals within the process. However, couples and team strategies also incorporate other approaches not available to individual performers. For example, they can create a supportive culture that values *effective communication, mutual support, group problem solving*, and *shared goal setting*. In doing so, they improve the likelihood of better outcomes and diminish the individual burden of failure and/or negative evaluation, as the responsibility for high performance (or failure) is distributed across the partnership or the team.

## 5. Conclusions and Future Directions

The use of the RIM framework could simultaneously improve understanding and prediction of performance-enhancing interventions as well as accelerate the advance-ment of performance-related strategies in Sex, Sport, and Stage. Researchers might consider how the variables they study could fit within particular RIM processes with its sample-nested factors (Figure 1 and Figure 2) and, based on the four draft goals of interventions outlined previously, how they might preserve or enhance automatic processing in high performance demand situations. Building an intricate and unifying model around RIM could help identify new connections among functions, mediating/moderating variables responsible for improvement, and gaps in both research and application. It would enable development of intervention models that test the role of various factors both within and across domains, and help identify remedial procedures that complement one another by addressing the different intervention points related to the deterioration of automatic processing dominance.

Regarding research designs, both quantitative and qualitative methods could ad-vance our understanding, prediction, and control within the RIM framework. Quantitative investigations employing between-group, within-group, and single subject methodologies can yield distinct yet overlapping knowledge. As performance, anxiety, and cognitive processing are likely multiply determined, multivariate models such as structural equation modeling, path analytics, and latent classification analyses could help identify potential causal (vs. correlational) relationships, moderating and mediating variables, and magnitude of effects. Eventually, meta-analyses or systemic reviews could reveal robust, additional, and unexpected relationships. Qualitative studies would provide a different lens to understanding these interconnections or aspects of them. For example, they might tap the performers’ experiences of various interventions as well as their experiences of working with intervention providers, or they might shed light on the subjective experiences of vacillation between automatic and reflective dominance over the course of implementation of deliberate practice.

## Figures and Tables

**Table 1:** Comparisons of common and domain-specific interventions strategies across Sex, Sport, and Stage.

Domain	Sex	Sport	Stage
**Shared Interventions**			
Cognitive	Reframing; positive self-talk; identifying and replacing faulty beliefs/coping; maintaining focus on relevant stimuli; improving attitude, self-efficacy, confidence.	Reframing; restructuring; positive self-talk; moderating expectations; thinking more productively; trust monitoring; attentional control; dual task training; coaching-led intervention.	Reframing; positive self-talk; problem focused coping strategies; positive self-efficacy; task related and emotion focused coping; modifying view of risk.
Mindfulness	Non-judgmental focus on thoughts, reactions, and emotions; relaxation; deep breathing; sensate focus—awareness of body sensation; reduce performance demand.	Use of mental images to relax; employing other relaxation techniques; quiet eye training.	Imagery; relaxation; breathing; being in the moment; yoga exercises and meditation; stress reduction.
Emotion-focused	Increasing emotional awareness and regulation; reducing focus on fear/anxiety; replacing negative with positive emotion; productive channeling.	Adapting to pressure; performing under pressure; facilitative anxiety interpretation; countering negative self-talk.	Countering anxious habits; systematic desensitization; imagery; reinterpreting anxiety as facilitative.
Behavioral	Re-conditioning; altering stimuli and modifying responses; regulating arousal through use of stimuli.	Preparation; quiet eye training; pre-shot routines; controlling the controllables; mental/physical pre-performance routines; performing under pressure.	Practice strategies; rehearsal, controlling the controllables; using confidence stimulus; changing problematic behaviors stemming from anxiety.
General lifestyle/perspective	Emphasis on reducing lifestyle factors that increase risk of sexual problem (e.g., smoking). Expanding identity beyond sexual self.	Emphasis on healthy lifestyle; nutrition; proper care of body; self-regulation; energy management. Expanding identity beyond athlete.	Emphasis on healthy lifestyle, nutrition, proper care of body; self-regulation; energy management. Expanding identity beyond performer.
**Related or Secondary Approaches**			
Couples/team approaches	Diminishing evaluative component; couple working as a team; shared problem-solving; removing shame.	Developing high performance teams; culture of helping; shared problem solving and goal setting.	Using peer social support; developing shared strategies with colleagues; culture of helping; shared problem solving and goal setting.
Personality traits/dispositions	Mitigating negative traits and dispositions with cognitive approaches designed to counter perfectionism, lack of confidence, high self-consciousness, etc.	Mitigating negative traits and dispositions with cognitive approaches designed to counter perfectionism, lack of confidence, high self-consciousness, etc.	Mitigating negative traits and dispositions with cognitive approaches designed to counter perfectionism, lack of confidence, high self-consciousness, etc.
**Domain-Specific Strategies**			
Physio-pharmacological	Increasing or decreasing autonomic motor response via medication.		Using medication to alleviate autonomic symptoms thereby reducing anxiety triggers.
Preparation/practice		Pre-performance routines; rehearsals and practice; controlling the controllables.	Pre-performance routines; rehearsals and practice; controlling the controllables
Specific behavioral approaches		Using biofeedback and feedback from teammates and coach/director; reprogramming and correction of responses.	Using feedback from teammates and coach/director; reprogramming and correction of responses.
